# One new species of the genus *Savarna* Huber, 2005 (Araneae, Pholcidae) from southern Thailand

**DOI:** 10.3897/zookeys.498.9269

**Published:** 2015-04-21

**Authors:** Prasit Wongprom, Decha Wiwatwitaya

**Affiliations:** 1 Faculty of Forestry, Kasetsart University, Ladyao, Jatujak, Bangkok 10903, Thailand

**Keywords:** Taxonomy, morphology, pholcidae, southeast Asia

## Abstract

One new species *Savarna kraburiensis* **sp. n.** (♂♀) is reported from southern Thailand.

## Introduction

The small genus *Savarna* Huber, 2005 only contains three species: *Savarna baso* (Roewer, 1963) from Sumatra, Indonesia, *Savarna tesselata* (Simon, 1901) from Malaysia, and *Savarna thaleban* Huber, 2005 from Thailand (World Spider Catalog 2014). In this paper, we describe one more, a new species from Ranong, Thailand.

## Material and methods

Specimens were examined and measured with a Leica M205 C stereomicroscope; details were studied with an Olympus BX51 compound microscope. Male and female copulatory organs were examined and illustrated after they were dissected from the spiders. Epigynes were removed and treated in 10% warm solution of potassium hydroxide (KOH) before illustration. Type specimens were preserved in 75% ethanol solution. Photographs were taken with an Olympus C7070 wide zoom digital camera (7.1 megapixels) mounted on a Leica M205 C stereomicroscope. The images were assembled using Helicon Focus 3.10 image stacking software. All measurements are given in millimeters unless noted otherwise. Leg measurements are shown as: Total length (femur + patella + tibia + metatarsus + tarsus). Leg segments were measured on their dorsal side. Type specimens are deposited in the Thailand Natural History Museum, Pathum Thani, Thailand.

Terminology and taxonomic descriptions follow Huber (2000). The following abbreviations are used in the descriptions: ALE = anterior lateral eye, AME = anterior median eye, PME = posterior median eye, L/d = length/diameter.

## Taxonomy

### Genus Savarna

Taxon classificationAnimaliaAraneaePholcidae

Huber, 2005

#### Type species.

*Savarna thaleban* Huber, 2005

### Savarna kraburiensis sp. n.

Taxon classificationAnimaliaAraneaePholcidae

http://zoobank.org/ADD9975B-7C08-493B-8D8C-9CEE4CE3A4F3

Figs 1 , 2

#### Type material.

Holotype: ♂, near the entrance of Phra Kha Yang Cave (10°19.568'N, 98°45.908'E, elevation 6 m), Kraburi District, Ranong, Thailand, 28 October 2014, P. Wongprom leg. Paratypes: 1♂, 2♀♀, same data as holotype.

#### Etymology.

The specific name refers to the type locality; adjective.

#### Diagnosis.

**Figure 1.**
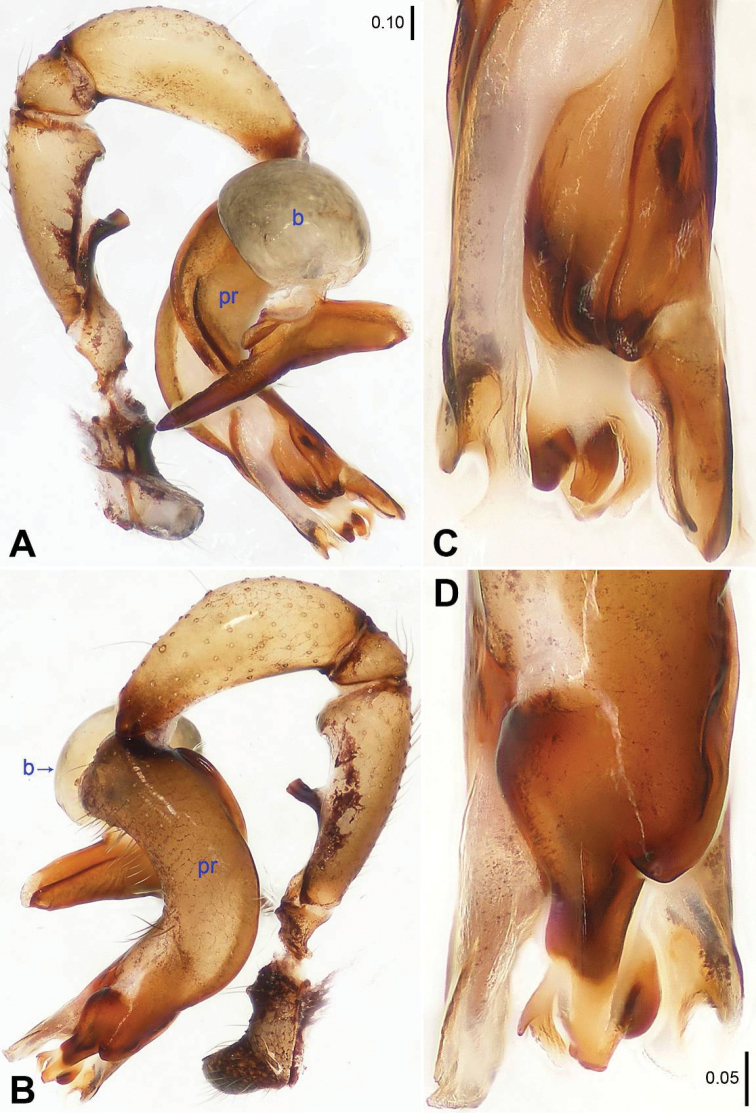
*Savarna kraburiensis* sp. n., holotype male. **A–B** Pedipalp (**A** Prolateral view **B** Retrolateral view) **C–D** Distal part of procursus (**C** Prolateral view **D** Retrolateral view). b = bulb, pr = procursus.

**Figure 2.**
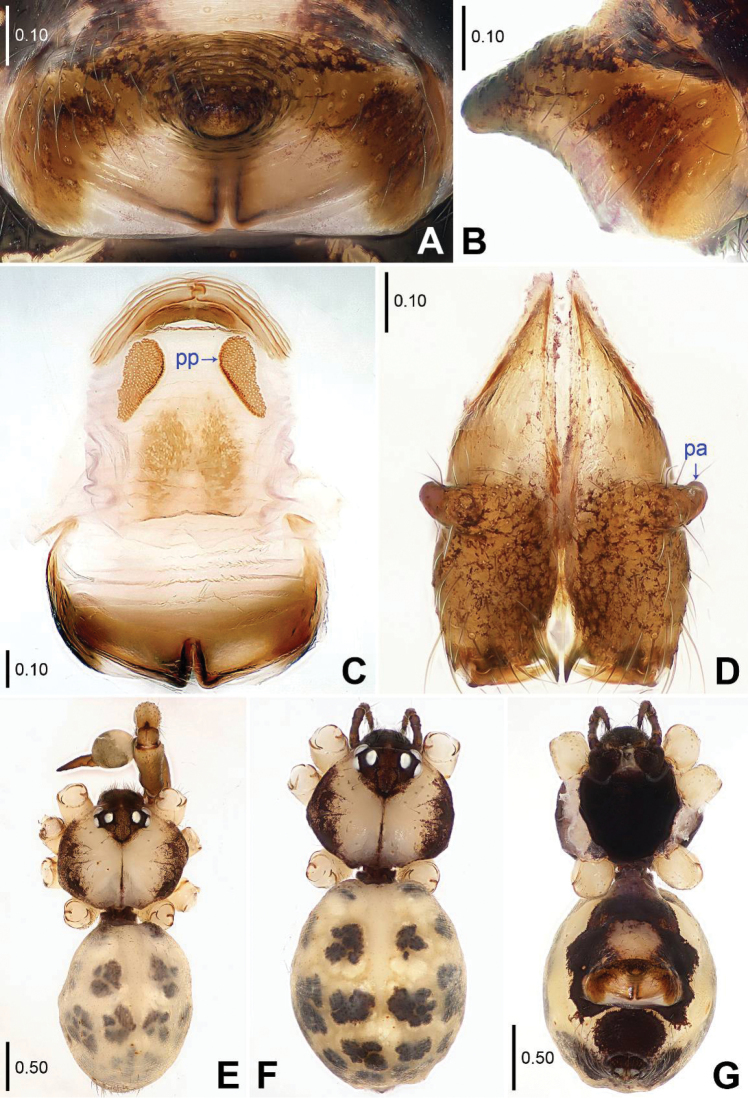
*Savarna kraburiensis* sp. n., holotype male (**D–E**) and paratype female (**A–C, F–G**). **A–B** Epigyne (**A** Ventral view **B** Lateral view) **C** Vulva, dorsal view **D** Chelicerae, frontal view **E–G** Habitus (**E–F** Dorsal view **G** Ventral view). pa = proximo-lateral apophysis, pp = pore plate.

The species resembles *Savarna tesselata* (Simon, 1901) (see Huber 2005: 78, figs 129–130, 138–140), but can be distinguished by absence of median apophyses on male clypeus (Fig. 2E), by relatively wide pedipalpal tibia subproximally (Figs 1A–B), and by shape of bifurcated distal apophysis on bulb (Fig. 1A).

#### Description.

**Male (holotype).** Total length 2.97 (3.13 with clypeus), prosoma 1.04 long, 1.34 wide, opisthosoma 1.93 long, 1.44 wide. Leg I: 27.73 (7.63 + 0.55 + 7.05 + 9.94 + 2.56), leg II: 18.52 (5.45 + 0.48 + 4.62 + 6.50 + 1.47), leg III: 13.52 (4.04 + 0.47 + 3.27 + 4.77 + 0.97), leg IV: 18.36 (5.64 + 0.48 + 4.49 + 6.73 + 1.02). Habitus as in Fig. 2E. Dorsal shield of prosoma yellowish, with black margins and a narrow, dark median line behind ocular area; sternum black. Legs brownish, but slightly whitish on femora (distally) and tibiae (distally), with slightly darker rings on femora (subdistally). Opisthosoma yellowish, with black spots. Distance PME-PME 0.20, diameter PME 0.12, distance PME-ALE 0.04, AME absent. Ocular area slightly elevated and separated from rest of prosoma. Thoracic furrow distinct and deep. Sternum slightly wider than long (0.87/0.78). Chelicerae as in Fig. 2D, with a pair of proximo-lateral apophyses. Pedipalps as in Figs 1A–B; trochanter with a curved ventral apophysis lying against femur; procursus simple proximally but complex distally; bulb with a proximal sclerite and a bifurcated distal apophysis. Retrolateral trichobothrium of tibia I at 9%; legs with short vertical hairs on tibiae, without spines and curved hairs; tarsus I with more than 30 distinct pseudosegments.

**Variation.** Tibia I in another male: 6.73.

**Female.** Similar to male, habitus as in Figs 2F–G. Tibia I (n=2): 6.22, 6.35. One of the specimens measured: Total length 2.69 (2.81 with clypeus), prosoma 0.89 long, 1.13 wide, opisthosoma 1.80 long, 1.47 wide; tibia I: 6.22. Distance PME-PME 0.18, diameter PME 0.12, distance PME-ALE 0.03, AME absent. Epigyne (Figs 2A–B) strongly protruding, without pockets. Vulva (Fig. 2C) with a pair of pore plates.

#### Distribution.

Known only from the type locality.

## Supplementary Material

XML Treatment for Savarna

XML Treatment for Savarna kraburiensis
